# Boosting Photocatalytic Performance of ZnO Nanowires via Building Heterojunction with g-C_3_N_4_

**DOI:** 10.3390/molecules28145563

**Published:** 2023-07-21

**Authors:** Yayang Wang, Ziyi Liu, Yuesheng Li, Xiaojie Yang, Lingfei Zhao, Jian Peng

**Affiliations:** 1 Hubei Key Laboratory of Radiation Chemistry and Functional Materials, School of Nuclear Technology and Chemistry & Biology, Hubei University of Science and Technology, Xianning 437100, China; wyy934313@163.com (Y.W.); yiyi3092208242@163.com (Z.L.); 2 School of Chemistry and Chemical Engineering, Wuhan University of Science and Technology, Wuhan 430074, China; 3 Institute for Superconducting and Electronic Materials, Australian Institute for Innovative Materials, Innovation Campus, University of Wollongong, Squires Way, North Wollongong, NSW 2522, Australia; lingfeiz@uow.edu.au

**Keywords:** g-C_3_N_4_, ZnO NW, heterostructure, photodegradation

## Abstract

The development of a stable and highly active photocatalyst has garnered significant attention in the field of wastewater treatment. In this study, a novel technique involving a facile stirring method was devised to fabricate an array of g-C_3_N_4_/ZnO nanowire (ZnO NW) composites. Through the introduction of g-C_3_N_4_ to augment the generation of electron-hole pairs upon exposure to light, the catalytic efficacy of these composites was found to surpass that of the pristine ZnO NWs when subjected to simulated sunlight. The photocatalytic performance of a 20 mg·L^−1^ methylene blue solution was found to be highest when the doping rate was 25 wt%, resulting in a degradation rate of 99.1% after 60 min. The remarkable enhancement in catalytic efficiency can be ascribed to the emergence of a captivating hetero-junction at the interface of g-C_3_N_4_ and ZnO NWs, characterized by a harmoniously aligned band structure. This alluring arrangement effectively curtailed charge carrier recombination, amplified light absorption, and augmented the distinct surface area, culminating in a notable boost to the photocatalytic prowess. These findings suggest that the strategic engineering of g-C_3_N_4_/ZnO NW heterostructures holds tremendous promise as a pioneering avenue for enhancing the efficacy of wastewater treatment methodologies.

## 1. Introduction

The swift growth of the socio-economy and chemical industry as resulted in the release of large volumes of wastewater containing antibiotics and dyes into water bodies, leading to water pollution. This poses a threat to ecosystems, human health, and the environment, making it a pressing issue that requires attention [1,2,3,4,5]. Over time, various methods for the removal of toxic components in wastewater have continued to be devised, including membrane separation, adsorption, chemical methods, and photocatalytic technology [6,7,8,9,10,11]. Among these techniques, semiconductor photocatalysis had emerged as one of the most crucial and promising method due to its ease of handling, good reproducibility, simplicity, outstanding cost-effective performance, and environmental friendliness [12,13]. Therefore, designing and developing a novel semiconductor material with broad spectral response, low cost, outstanding photoactivity, and excellent stability is a key research area in the quest to improve the efficient utilization of solar energy [14].

Previous studies had investigated numerous semiconductors as efficient photocatalysts, such as TiO_2_ [15], ZnO, SrTiO_3_ [16], Fe_2_O_3_ [17], CdS [18], WO_3_ [19], and ZnS [20]. Among numerous multifunctional semiconductor metal oxide materials, ZnO had been widely applied in fields such as luminescent materials, gas sensing, optical devices, solar cells, and photocatalysis due to its characteristics including non-toxicity, ease of substitution, low cost, and stability. As an emerging semiconductor material, ZnO had received significant attention worldwide for its potential in degrading organic pollutants and is generally considered one of the most attractive semiconductor materials [21]. While ZnO offers numerous benefits, its usefulness in practical applications is restricted by its wide band gap of 3.37 eV and high recombination efficiency of electron-hole pairs. To overcome these limitations, researchers had employed various strategies, such as doping, surface modification, and forming nanostructures to improve the optical and electronic characteristics of the materials [22]. So far, fabricating ZnO-based semiconductor composites has been proved to be an effective route to improving the photocatalytic activity of ZnO. By utilizing the difference between the conduction bands and valence of various photocatalytic materials, this approach successfully segregates photogenerated electrons and holes, thereby increasing the photocatalytic capability of ZnO. Moreover, semiconductors with narrow band gaps can absorb visible light, thereby expanding the light-absorption range of ZnO [23,24,25,26]. Li et al. developed the chemical vapor deposition method to synthesis ZnO nanowires (ZnO NWs) decorated with Au NPs on silicon substrates [27]. The photocatalytic activity of Au/ZnO NWs was found to be significantly higher than that of pristine ZnO NWs in the degradation of methylene blue (MB) solution under simulated sunlight irradiation. In order to improve the photocatalytic efficiency of ZnO NWs, Yamina et al. [28] successfully synthesized iron-doped ZnO NWs (Fe/ZnO NWs) by a two-step hydrothermal method. The Fe/ZnO NWs were tested for their photocatalytic ability to break down organic pollutants in water using simulated pollutants like MB, methyl orange (MO), and acid red 14 (AR14). The results showed that doping 1 wt% Fe/ZnO NWs led to a 9%, 20%, and 5% increase in photodegradation rates of MB, MO, and AR14, respectively. Z. Braiek et al. [29] synthesized ZnO/In_2_S_3_ core/shell NWs on indium tin oxide substrates through a simple and economical two-step electrochemical method, with successful results. Experimental results showed that the ZnO/In_2_S_3_ core/shell NWs could completely degrade pollutants within 120 min, with photodegradation rates that were five times faster than those of pure ZnO NWs. Nevertheless, the existing precursor selections in these state-of-the-art methods remain inadequate for the practical implementation of ZnO-based catalysis in wastewater treatment. Major hurdles include their exorbitant costs, intricate synthesis pathways, and inherent toxicity. Thus, the development of innovative synthesis techniques becomes imperative in order to fabricate ZnO-based catalytic materials that can foster the advancement of low-cost, highly efficient, and safe methods for wastewater purification.

Although the heterojunction between ZnO and g-C_3_N_4_ is not new, it is still of paramount importance in developing a simple, efficient, and low-cost method to synthesize a high-stability and high-performance catalyst. In this work, a remarkable set of g-C_3_N_4_/ZnO NW composites, showcasing exceptional catalytic performance, remarkable stability, and effortless recyclability, were successfully synthesized through the meticulous selection of precursors and the implementation of a novel and straightforward stirring technique. In the preparation process of ZnO NWs, the samples demonstrated a remarkable level of reproducibility. This could be attributed to several factors, including the simplicity of the operation, meticulous control over specific experimental conditions and relevant parameters, and the excellent reproducibility of the synthesis method employed. Additionally, the stability and uniformity maintained throughout the synthesis process further contributed to the exceptional reproducibility observed in the prepared ZnO NW samples. Due to the slender band gap (E_g_ = 2.7 eV), ample specific surface area, robust photostability, and non-toxic, non-polluting nature of the elaborately selected g-C_3_N_4_, the as-prepared g-C_3_N_4_/ZnO NW composites showed enhanced photocatalytic performance. The crystal phase, morphology, chemical composition, valence, optical properties, and specific surface area of the samples were analyzed by multiple techniques. In addition, the photocatalytic performance of the prepared catalyst was evaluated through light-induced degradation experiments of MB. The stability of the catalyst was explored by cycling experiments and possible degradation mechanisms were proposed and discussed by analyzing active species trapping experiments. This study could provide a valuable roadmap for harnessing the potential of g-C_3_N_4_/ZnO semiconductor photocatalysts in the eradication of organic pollutants from wastewater, paving the way for groundbreaking advancements in the field of wastewater treatment.

## 2. Results and Discussion

### 2.1. XRD and FT-IR Analysis

**Figure 1:**
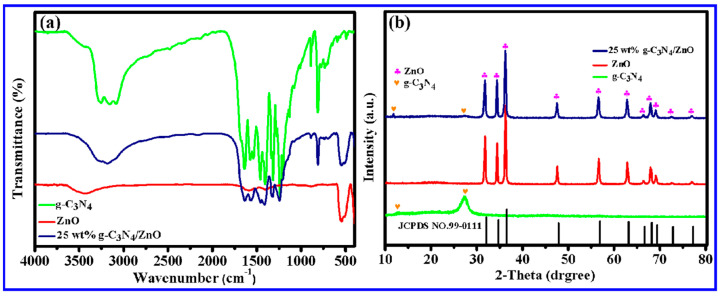
Characterization diagrams of g-C_3_N_4_, ZnO NWs, and 25 wt% g-C_3_N_4_/ZnO NWs: (**a**) IR pattern and (**b**) XRD pattern.

Figure 1a presented the Fourier transform infrared (FT-IR) spectrum of the g-C_3_N_4_, ZnO NWs, and 25 wt% g-C_3_N_4_/ZnO NWs. The peaks observed at 1245.19 cm^−1^ and 1639.51 cm^−1^ in the g-C_3_N_4_ spectrum were attributed to the C-N and C=N stretching vibrations in the aromatic carbon–nitrogen heterocycles. Furthermore, a vibration peak originating from three-s-triazine rings appeared near 812.14 cm^−1^, which corresponded to the XRD results and provided further evidence of the graphene-like structure of g-C_3_N_4_. The relatively weaker absorption peak at 546.42 cm^−1^ could be assigned to the symmetric stretching vibration of Zn-O, indicating the formation of ZnO crystals. The FT-IR spectrum of 25 wt% g-C_3_N_4_/ZnO NWs was similar to the characteristic spectra of g-C_3_N_4_ and ZnO NWs, providing evidence that g-C_3_N_4_ was effectively composited with ZnO NWs while retaining its typical graphite structure.

Figure 1b shows the X-ray powder diffraction (XRD) patterns of the g-C_3_N_4_, ZnO NWs, and 25 wt% g-C_3_N_4_/ZnO NW composites. The XRD pattern of the g-C_3_N_4_ showed two obvious diffraction peaks at 13.0° and 27.3°. The diffraction peak near 13.0° belonged to the (100) plane and represented the distance of the repeating unit in the plane of the conjugated layer, corresponding to the macrocyclic structure between the three-s-triazine units. The characteristic peak near 27.3° could be attributed to the (002) plane of typical graphitic layered structure, which was attributed to the interlayer stacking structure of aromatic hydrocarbons [30]. The characteristic diffraction peaks of the ZnO NWs at 2θ of 31.77°, 34.42°, 36.25°, 47.54°, 56.59°, 62.85°, 66.37°, 67.94°, 69.08°, 72.56°, and 76.95° were assigned to (100), (002), (101), (102), (110), (103), (200), (112), (201), (004), and (202) crystal faces (JCPDS NO.99-0111), indicating that ZnO with wurtzite structure was successfully prepared. The XRD patterns of 25 wt% g-C_3_N_4_/ZnO NW composites exhibited the characteristic peaks of g-C_3_N_4_ and ZnO at the same time, and each diffraction peak was sharp without impurity peaks. The successful preparation of g-C_3_N_4_/ZnO NWs was confirmed, and it was also shown that the resulting samples exhibited excellent crystallinity.

### 2.2. XPS Analysis

**Figure 2:**
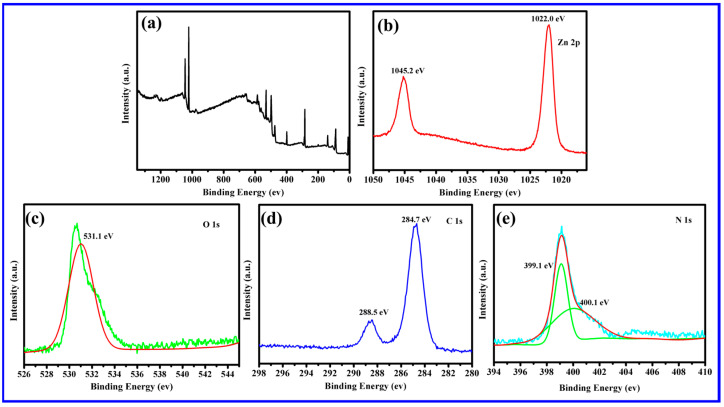
XPS spectra of 25 wt% g-C_3_N_4_/ZnO NWs: (**a**) survey spectrum, (**b**) Zn 2p, (**c**) O 1s, (**d**) C 1s, and (**e**) N 1s.

XPS measurements were conducted to analyze the superficial composition and chemical state of the samples (Figure 2). The full survey spectrum in Figure 2a confirmed the presence of Zn, O, C, and N elements in the 25 wt% g-C_3_N_4_/ZnO NW composite, providing evidence for the co-existence of 25 wt% g-C_3_N_4_ and ZnO NWs in the composite. As reported previously [31], the two sharp peaks at 1045.2 eV (Zn 2p_1/2_) and 1022.0 eV (Zn 2p_3/2_) in Figure 2b matched well with that of Zn^2+^. In Figure 2c, the characteristic peak of O 1s at 531.1 eV corresponded to O^2−^ in ZnO. The observed binding energies at 284.7 eV and 288.5 eV in the C 1s sub-peak spectrum of Figure 2d corresponded to the C-C and C=N bonds, respectively. In Figure 2e, the strong characteristic peaks of N 1s appeared at 399.1 eV and 400.1 eV, corresponding to C-N=C and N-(C)_3_, respectively, which further verified the successful composite of ZnO NWs and g-C_3_N_4_. The XPS results provided additional evidence to support the successful formation of the heterojunction, which was consistent with the other characterization findings.

### 2.3. Morphology and Microstructure Analysis

**Figure 3:**
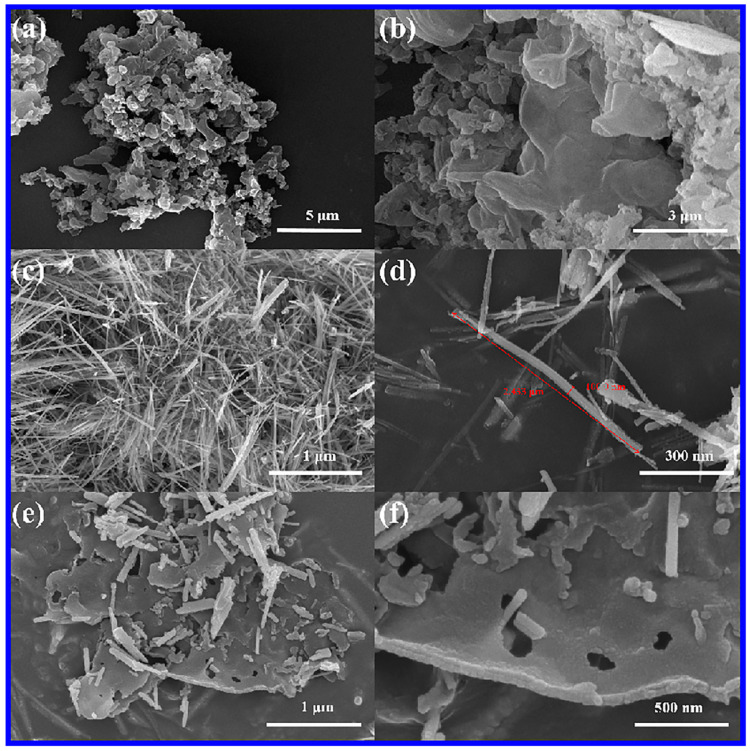
(**a**,**b**) SEM images of g-C_3_N_4_, (**c**,**d**) SEM images of ZnO NWs, and (**e**,**f**) SEM images of 25 wt% g-C_3_N_4_/ZnO NWs.

**Figure 4:**
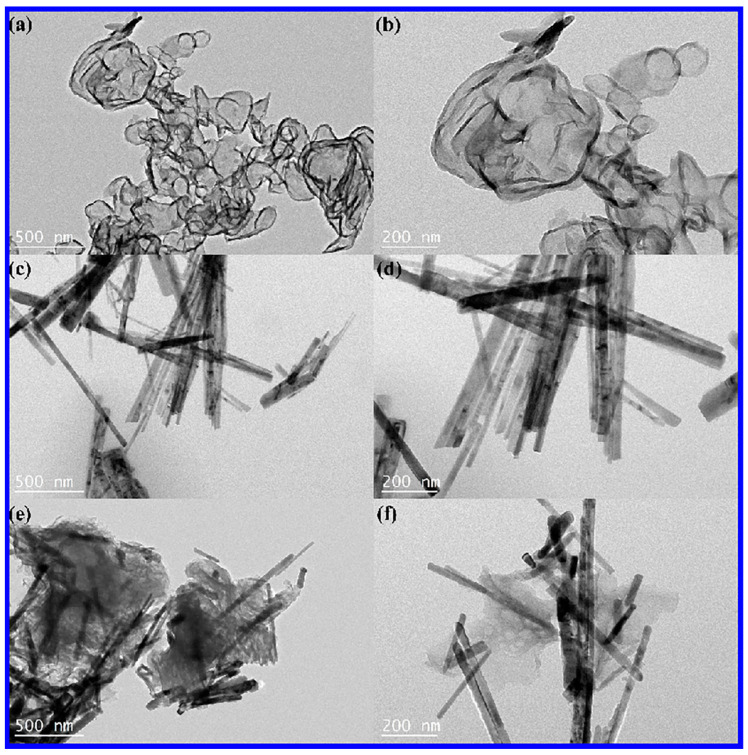
(**a**,**b**) TEM images of g-C_3_N_4,_ (**c**,**d**) TEM images of ZnO NWs, and (**e**,**f**) TEM images of 25 wt% g-C_3_N_4_/ZnO NWs.

The morphology of g-C_3_N_4_, ZnO NWs, and 25 wt% g-C_3_N_4_/ZnO NW samples was analyzed by scanning electron microscope (SEM). Figure 3a,b show the multilayer stacked structure of g-C_3_N_4_. As shown in Figure 3c,d, the prepared ZnO NWs exhibited a linear structure with a diameter of approximately 100 nm and a length of approximately 2.433 × 10^3^ nm. The surface of these nanowires presented fine and dense features, without any apparent orientation or pointed structure. Figure 3e,f clearly indicate that, in addition to the stacking of g-C_3_N_4_ sheets in 25 wt% g-C_3_N_4_/ZnO NWs, the linear structure of ZnO was uniformly distributed on the surface of g-C_3_N_4_. This structure could be beneficial to the formation of heterojunctions to enhance the photocatalytic activity. The lamellar structure of g-C_3_N_4_, the disordered linear structure of ZnO NWs, and the microstructure of g-C_3_N_4_/ZnO NW composites were further analyzed by transmission electron microscopy. The results of both Figure 3 and Figure 4 illustrate that ZnO was dispersed on the surface of g-C_3_N_4_, which was in line with the findings of XRD and SEM analyses.

### 2.4. Analysis of Optical Properties

**Figure 5:**
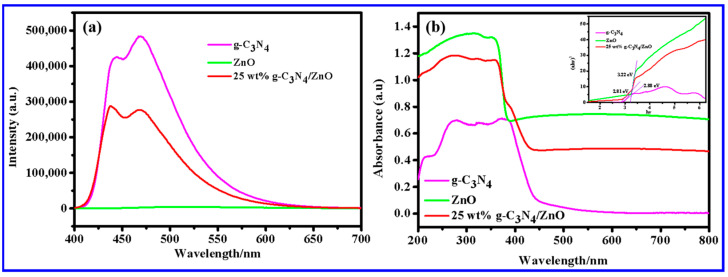
(**a**) Photoluminescence (PL) emission spectra of g-C_3_N_4_, ZnO NWs, and 25 wt% g-C_3_N_4_/ZnO NWs and (**b**) UV-Vis spectra of g-C_3_N_4_, ZnO NWs, and 25 wt% g-C_3_N_4_/ZnO NWs.

In order to assess the rate of recombination of photoinduced charge carriers, photoluminescence (PL) emission spectra were measured. The optical properties of g-C_3_N_4_, ZnO NWs, and 25 wt% g-C_3_N_4_/ZnO NWs were investigated with an excitation wavelength of 365 nm. As shown in Figure 5a, the PL intensity of 25 wt% g-C_3_N_4_/ZnO NWs was much weaker than that of single g-C_3_N_4_. This result indicated that the recombination rate of photoinduced carriers in the 25 wt% g-C_3_N_4_/ZnO NW composite was greatly reduced, thereby enhancing the photocatalytic activity.

The optical properties of the photocatalyst were analyzed by UV-Vis DRS. Figure 5b shows the UV-Vis spectra and bandgap diagrams (upper right inset) of g-C_3_N_4_, ZnO NWs, and 25 wt% g-C_3_N_4_/ZnO NWs, with E_g_ values of 2.88 eV, 3.22 eV, and 2.81 eV, respectively. The successful recombination of g-C_3_N_4_ with ZnO NWs resulted in a narrow band gap and better photo response.

### 2.5. N_2_ Adsorption–Desorption Isotherm Analysis

**Figure 6:**
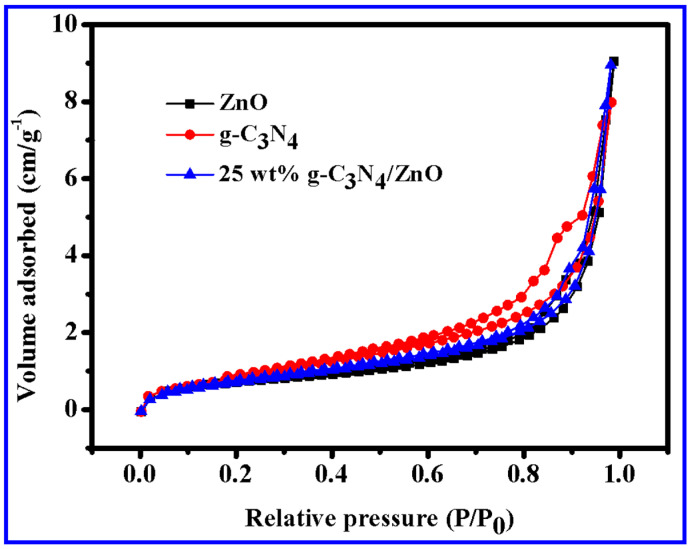
Nitrogen multilayer adsorption–desorption curves of g-C_3_N_4_, ZnO NWs, and 25 wt% g-C_3_N_4_/ZnO NWs.

The surface area of the catalysts was measured by testing their specific surface area. The nitrogen adsorption–desorption isotherms for three different samples are presented in Figure 6, including g-C_3_N_4_, ZnO NWs, and a combination of 25 wt% g-C_3_N_4_/ZnO NWs. According to the data, the measured specific surface areas of g-C_3_N_4_, ZnO NWs, and 25 wt% g-C_3_N_4_/ZnO NWs were 22.5233 m^2^·g^−1^, 10.2677 m^2^·g^−1^, and 15.4558 m^2^·g^−1^, respectively. The analysis of the data showed that the specific surface area of ZnO NWs was significantly increased after g-C_3_N_4_ was combined with ZnO NWs. As the specific surface area increases in a photocatalytic reaction, contact with the reactants can be achieved more fully. This is highly beneficial for the later stages of the photocatalytic process.

### 2.6. Evaluation of Photocatalytic Degradation Performance

**Figure 7:**
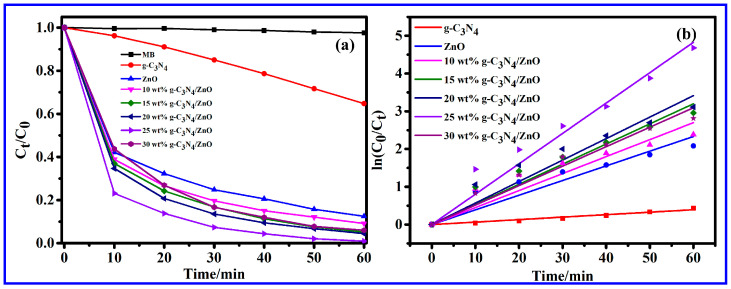
(**a**) Photocatalytic degradation curves of MB on different prepared samples and (**b**) degradation kinetics curves of MB with different photocatalysts.

MB was used as a simulated pollutant to explore the photocatalytic performance of the catalyst under visible light irradiation (λ ≥ 400 nm). To ensure that MB did not undergo self-degradation, a blank control experiment was incorporated in the study. Specifically, 20 mg·L^−1^ MB was subjected to photodegradation without the presence of any catalyst. Based on the subsequent experimental findings, it can be concluded that MB does not possess the ability to self-degrade and is suitable for use in subsequent photocatalytic degradation experiments. It could be seen from Figure 7a that the photocatalytic performance of g-C_3_N_4_/ZnO series composites was enhanced when compared with single ZnO. The activity of the photocatalysts followed the order g-C_3_N_4_/ZnO NWs, pure ZnO NWs, and g-C_3_N_4_. When the doping ratio of g-C_3_N_4_ was 25 wt%, the formed composite material showed the best performance in degrading MB, and the degradation rate of MB solution was as high as 99.07% within 60 min. The g-C_3_N_4_/ZnO NW composite exhibited excellent photocatalytic performance, which can be attributed to two factors. Firstly, the combination of g-C_3_N_4_ and ZnO NWs increased light absorption and specific surface area. Secondly, the formation of the heterostructure facilitated the transfer and separation of charge carriers. The results further indicated that the as-prepared composite was of high photocatalytic activity and potential ability to efficiently degrade organic pollutants.

In order to gain a deeper understanding of the mechanism in the photocatalytic degradation activity of various catalysts for MB, the experimental data in Figure 7a were fitted with the first-order kinetics according to the formula −ln (*C_t_*/*C*_0_) = k*t*, as shown in Figure 7b. In this formula, “*C*_0_” refers to the initial concentration of MB, “*C_t_*” represents the concentration of MB after a given illumination time “*t*”, and “k” denotes the rate constant of pseudo first order. By plotting the graph of −ln (*C_t_*/*C*_0_) versus time “*t*”, the rate constants for all photocatalysts were determined from the slope of the straight line. Table 1 lists the corresponding kinetic rate constants and the corresponding MB degradation rates when using different catalysts. From the analysis of the data, it is worth noting that all samples exhibited an almost linear curve (R^2^ > 0.96), which confirmed that the degradation of MB dye followed pseudo-first-order reaction kinetics. It is well-known that a higher photocatalytic activity is achieved with a higher first order constant [32]. The catalytic reaction rate of the composite catalysts was higher than that of pure ZnO. Moreover, the k value of 25 wt% g-C_3_N_4_/ZnO was the largest (0.08045), and 25 wt% g-C_3_N_4_/ZnO also produced the best degradation effect, which is consistent with the experimental results.

### 2.7. Reusability and Stability of the Composites

**Figure 8:**
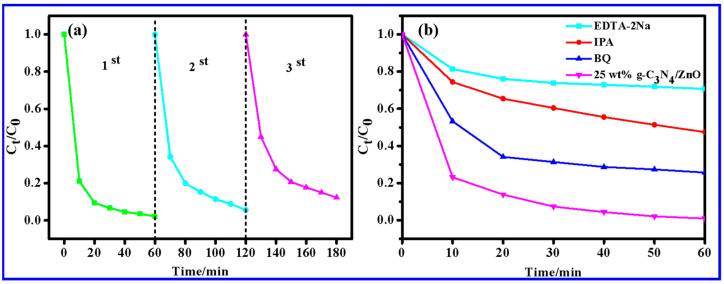
MB degradation by 25 wt% g-C_3_N_4_/ZnO NWs: (**a**) cycle experiment diagram and (**b**) capture experiment diagram of related active substances.

Apart from exhibiting excellent photocatalytic performance, assessing the reusability and cycle stability of photocatalysts is a necessary and crucial step in their practical application. Therefore, 25 wt% g-C_3_N_4_/ZnO NWs were subjected to continuous degradation of MB to evaluate their recovery availability under identical conditions. After each cycle test, the catalyst was first subjected to high-speed centrifugation, followed by several washes with ethanol and water. Finally, the catalyst was dried in an oven at 60 °C, ground, and reused in the next cycle. As shown in Figure 8a, the catalytic degradation efficiency for MB of 25 wt% g-C_3_N_4_/ZnO NWs decreased slightly when the number of degradation cycles increased. The photocatalyst was tested for reusability and cycle stability by conducting three cycle experiments, where 25 wt% g-C_3_N_4_/ZnO NWs were continuously used to degrade MB under the same conditions. After each cycle, the used catalyst was centrifuged, washed with ethanol and water, dried at 60 °C, and ground before reuse. The results showed that the degradation rates of MB by the photocatalyst were 97.77%, 94.44%, and 87.67% in the first, second, and third cycles, respectively. Despite this gradual decline in performance, the removal efficiency of MB remained high at 80.2% after three degradation−regeneration runs, indicating the good reusability and cycle stability of the composite photocatalyst.

### 2.8. Possible Photodegradation Mechanism

To identify the active species involved in the photocatalytic process, radical species trapping experiments were carried out by adding quenchers, including EDTA-2Na (h^+^ scavenger), IPA (·OH scavenger), and BQ (·O^2−^ scavenger). As shown in Figure 8b, the photocatalytic performance of 25 wt% g-C_3_N_4_/ZnO NW was greatly affected by the addition of EDTA-2Na. Its degradation rate decreased from 99.07% to 29.34%, indicating that h^+^ was the main contributor to MB degradation. Adding IPA inhibited the degradation of MB to some extent, and the degradation rate was reduced from 99.07% to 52.53%, indicating that ·OH also participated in the photocatalytic reaction process. The introduction of BQ as a capture agent for ·O^2−^ had little effect on MB degradation, and the degradation rate decreased from 99.07% to 74.32%. Therefore, ·OH and h^+^ were the main active species of the 25 wt% g-C_3_N_4_/ZnO NWs in the catalytic degradation of MB under visible light.

**Figure 9:**
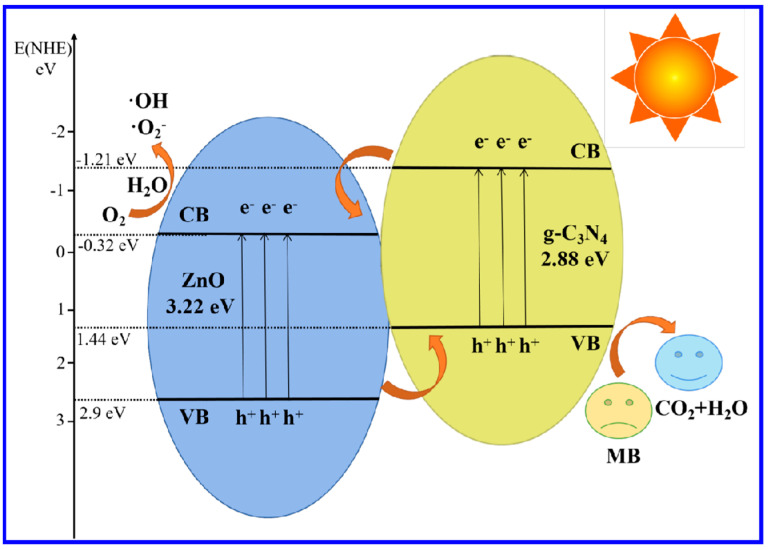
Schematic diagram for the proposed photocatalytic reaction mechanism of 25 wt% g-C_3_N_4_/ZnO NWs.

Based on the relevant characterization data and experimental results, Figure 9 reveals the possible mechanism of complex photocatalytic degradation of MB by 25 wt% g-C_3_N_4_/ZnO NWs. Since the band gaps of g-C_3_N_4_ and ZnO NWs were 2.88 eV and 3.22 eV, respectively, the valence and conduction band positions of g-C_3_N_4_ and ZnO NWs were roughly calculated from the forbidden band width E_g_. The CB potential values of g-C_3_N_4_ and ZnO NWs were −1.21 eV and −0.32 eV, respectively, and the VB potential values of g-C_3_N_4_ and ZnO NWs were 1.44 eV and 2.9 eV, respectively. Both the CB potential and VB potential values of g-C_3_N_4_ were more negative than those of ZnO NWs, therefore, this potential difference enabled the formation of a unique heterostructure inside the composite. Under simulated sunlight illumination, both g-C_3_N_4_ and ZnO NWs were activated and generated photogenerated electron-hole pairs. The e^−^ on the CB of g-C_3_N_4_ migrated rapidly to the CB of ZnO, while the h^+^ of VB in ZnO migrated to the VB potential of g-C_3_N_4_. This well-matched energy-band structure was conducive to the effective separation of photogenerated electron-hole pairs between g-C_3_N_4_ and ZnO NWs, which improved the photocatalytic activity of g-C_3_N_4_/ZnO NW composites [33].

Compared to O_2_/·O^2−^ with a reduction potential of −0.33 eV/NHE, the CB position of g-C_3_N_4_ was more negative than O_2_/·O^2−^ and the potential of CB of ZnO NWs was more positive. Therefore, the e^−^ at the CB position of g-C_3_N_4_ could easily react with dissolved oxygen to form ·O^2−^ while the e^−^ at the CB position of ZnO NWs could not reduce O_2_ to ·O^2−^. Compared to O_2_/H_2_O_2_ with a reduction potential of 0.695 eV/NHE, the e^−^ at the CB position of ZnO NWs could react with O_2_ and h^+^ in water to form ·OH. The VB of g-C_3_N_4_ was lower than that of OH/H_2_O (+2.72 eV/NHE), so the h^+^ on the VB of g-C_3_N_4_ could not oxidize H_2_O to ·OH and directly react with MB. However, the potential of ZnO NWs was higher than that of the OH/H_2_O, so the h^+^ on its CB could oxidize H_2_O to ·OH, which could also be used in the following oxidation reaction. ·OH, h^+^, and ·O^2−^ free-radical active substances will undergo redox reaction with MB, and then be degraded into small molecular organic compounds, and finally be degraded into non-toxic and harmless H_2_O and CO_2_ [34].

## 3. Experimental

### 3.1. Materials

Zinc acetate dihydrate (CH_3_COO)_2_Zn·2H_2_O), urea (CN_2_H_4_O), isopropanol (C_3_H_8_O, IPA), disodium edetate (C_10_H_14_Na_2_O_8_, EDTA-2Na), potassium bromide (KBr), and absolute ethanol (C_2_H_6_O) were purchased from Shanghai Sinopharm Chemical Reagent Co., Ltd. Shanghai, China. Methylene blue (C_16_H_18_N_3_ClS, MB) and p-benzoquinone (C_6_H_4_O_2_, BQ) were purchased from Shanghai Aladdin Biochemical Technology Co., Ltd. Shanghai, China. The chemical reagents used in this experiment were of analytical grade and were not further purified before use.

### 3.2. Preparation of g-C_3_N_4_

We weighed 70 g of urea (CH_4_N_2_O) into an alumina crucible, and transferred the covered crucible to a muffle furnace. Calcination was performed at a constant temperature of 550 °C for 180 min, and the heating rate was set at 5 °C·min^−1^. The sample was calcined and grounded to obtain light-yellow g-C_3_N_4_ powder.

### 3.3. Preparation of ZnO NWs

ZnO NWs were prepared by direct solid-state thermal decomposition of (CH_3_COO)_2_Zn 2H_2_O. We took a 14 g sample of (CH_3_COO)_2_Zn 2H_2_O and ground it in an agate mortar for 10 min, then transferred it to a 30 mL alumina crucible. The crucible was placed in a muffle furnace and heated under an atmosphere of air to a constant temperature of 300 °C for 120 min. The heating rate was set to 2 °C·min^−1^. After the muffle furnace was cooled to room temperature, the gray ZnO NW powder was taken out, and the ZnO NWs were fully ground for later use.

### 3.4. Preparation of g-C_3_N_4_/ZnO NWs

We weighed 50 mg of g-C_3_N_4_ and added it to a reagent bottle containing the desired mass of ZnO NWs (the doping amounts of g-C_3_N_4_ were 10 wt%, 15 wt%, 20 wt%, 25 wt%, and 30 wt%), and added 40 mL of absolute ethanol and magnetons to the reagent bottle. After stirring for 8 h, the mixture was transferred to an oven at 60 °C for 48 h to remove absolute ethanol. The resulting solid was ground for later use.

### 3.5. Characterization

Fourier transform infrared (FT-IR) spectra were acquired using a spectrometer (Nicolet 5700, Thermo Fisher Scientific, MA, USA) to verify the presence and identify the specific vibration modes associated with functional groups. The sample’s crystal structure was determined using X-ray diffraction (XRD, Lab X XRD-6100, Shimazdu, Kyoto, Japan) with Cu-Kα radiation source. The measurements were taken with a working voltage of 40 kV and working current of 20 mA, and the scanning range spanned 2θ = 10~80°. The shapes and structures of the materials were examined using advanced imaging techniques, including field-emission scanning electron microscopy (FE-SEM, SU8220, Hitachi, Tokyo, Japan) and field emission transmission electron microscopy (FE-TEM, FEI Tecnai G2 F30, Hillsboro, OR, USA). X-ray photoelectron spectrometer (XPS) technology (Thermo ESCALAB 250XI, Shanghai Yuzhong Industrial Co., Ltd., Shanghai, China) was used to obtain the relevant information of the constituent elements, valence states, and chemical bonds of the samples. The binding energies of the elements in the samples were all based on the C 1s binding energy (284.6 eV) as the corrected binding energy. The Brunauer−Emmett−Teller method was used to measure the specific surface areas (BET, NOVA TOUCH LX1, Quantachrome, FL, USA). Ultraviolet-visible diffuse reflectance spectra were carried out by the spectrophotometer (UV-Vis DRS, UV-3600i Plus, Shimadzu Corporation, Kyoto, Japan) in the range of 200–800 nm. The Edinburgh FLS1000 fluorescence spectrophotometer was used to obtain the photoluminescence spectra of the samples at room temperature with the excitation laser wavelength of 365 nm. Additionally, the ultraviolet-visible (UV-Vis) spectrophotometric method (TU-1950, Persee, Nanjing, China) was employed to study the dye degradation.

### 3.6. Photocatalytic Experiment

The ability of a photocatalyst to decompose MB under visible light was used as a measure of its photocatalytic activity. All relevant experiments in this work were carried out in an open system at room temperature. In order to confirm that MB did not have the possibility of self-degradation, a blank control experiment was added. In the absence of a catalyst, the control experiment was carried out on the initial concentration of 20 mg·L^−1^ of MB. The detailed steps were as follows: 50 mg of photocatalyst was added to 50 mL MB solution with an initial concentration of 20 mg·L^−1^, and gently stirred for 30 min in the dark to obtain adsorption–desorption equilibrium. Next, using a xenon lamp as the light source, the photocatalytic degradation experiment was conducted under visible light irradiation, and 2 mL of the reaction solution was taken at regular intervals and placed it a centrifuge tube until the reaction was over. Finally, the centrifuge tube containing the reaction solution was placed in a centrifuge at 10,000 r·min^−1^, and the supernatant was taken after high-speed centrifugation. The absorbance values of MB (λ_max_ = 554 nm) supernatant under different reaction times were determined by UV-visible spectrophotometer. The degradation rate of MB (%) was calculated as follows:

$$\mathrm{Degradation}\;\mathrm{Rate}(\%)=\frac{C_{0}{-C}_{t}}{C_{t}}\times 100\%=\frac{A_{0}{-A}_{t}}{A_{0}}\times 100\%$$

where *C_t_* and *C*_0_ represented the concentration of MB corresponding to time *t* and *t*_0_, respectively, and *A*_0_ and *A_t_* represented the absorbance of the MB solution at time *t* and *t*_0_, respectively.

## 4. Conclusions

We have successfully designed a low-cost, intrinsically safe and facile “one-pot stirring” method to prepare a series of composite photocatalysts containing g-C_3_N_4_-doped ZnO NWs with enhanced photocatalytic performance by the meticulous selection of precursors. The photocatalytic degradation of MB was used to assess the catalytic performance of the photocatalyst under simulated sunlight irradiation. The 25 wt% g-C_3_N_4_/ZnO NWs showed the best photocatalyst performance, achieving an outstanding MB removal rate of 99.1% within 60 min. The improved performance of the composite could be attributed to several factors, including the improved separation of photogenerated electron-hole pairs, the large surface area providing more reaction sites, and the enhanced light-absorption capability. Furthermore, the composites exhibited excellent stability, maintaining their morphology, crystal phase, and catalytic activity after three cycles of testing. This work not only provides a more economical solution for the efficient purification of industrial wastewater, but also provides a theoretical basis for the study of ZnO-based composite materials, which is of great significance for opening up broad prospects for environmental protection applications.

## Figures and Tables

**Table 1:** Kinetic rate constants of MB degradation rate with different catalysts.

	Y = ln(*C*_0_/*C_t_*)	R^2^	Degradation Rate (%)
g-C_3_N_4_	Y = 0.00653X	0.98	35.21
ZnO	Y = 0.03886X	0.96	87.53
10 wt% g-C_3_N_4_/ZnO	Y = 0.04498X	0.96	90.82
15 wt% g-C_3_N_4_/ZnO	Y = 0.05332X	0.98	94.80
20 wt% g-C_3_N_4_/ZnO	Y = 0.05691X	0.98	95.55
25 wt% g-C_3_N_4_/ZnO	Y = 0.08045X	0.99	99.07
30 wt% g-C_3_N_4_/ZnO	Y = 0.05156X	0.98	93.99
